# Cell Surface Proteomics Analysis Indicates a Neural Lineage Bias of Rat Bone Marrow Mesenchymal Stromal Cells

**DOI:** 10.1155/2014/479269

**Published:** 2014-01-16

**Authors:** Guiyun Zhao, Huijiao Ji, Shihao Wang, Bin Gu, Xiuli Song, Jiarong Zhang, Yukan Liu, Liangbiao Chen, Ming Zhang

**Affiliations:** ^1^The Institute of Genetics, College of Life Sciences, Zhejiang University, Hangzhou 310058, China; ^2^Key Laboratory of Aquatic Resources and Utilization, Ministry of Education, College of Fisheries and Life Sciences, Shanghai Ocean University, Shanghai, 201306, China

## Abstract

Mesenchymal stromal cells (MSCs) are one of the most intensively studied stem cell types with application aims. However, the molecular characterisation and the relationship between the molecular characterisation and functional properties of MSCs are largely unknown. In this study, we purified the surface proteins from rat bone marrow MSCs (rBMMSCs) and characterised their surface proteome by LC-MS/MS. Moreover, we comparatively analysed the data from this study with the surface proteomics data of mouse and human embryonic stem (ES) cells and human mesenchymal stromal cells (hMSCs). The data showed that, in contrast to ES cells and human mesenchymal stromal cells, rBMMSCs possessed a surface proteomics pattern biased to neural and neural-endocrine lineages, indicating a neural/neural crest bias, and suggested a neural differentiation tendency of these cells. The different surface proteomics pattern between rBMMSCs and hMSCs also suggested that MSCs of different origin might possess a different lineage bias.

## 1. Introduction

Mesenchymal stromal cells are a type of widely distributed adult stem cell in connective tissues [1, 2]. MSCs are easy to isolate and propagate and possess the ability to differentiate into many different cell lineages, including osteoblasts, chondrocytes, adipocytes, hepatocytes, and neuron-like cells [3–6]. With these properties, MSCs have been widely applied in regenerative medicine research and experimental cell therapy for a wide spectrum of disorders such as neural injury [7], liver injury [8], and diabetes [9]. Moreover, recently several MSC-based clinical trials and therapeutics, such as Prochymal, have been approved by FDA [10, 11]. All of these facts suggest that MSCs are an important adult stem cell type, and a deep understanding of the molecular characteristics of MSCs would significantly promote the advancement of regenerative medicine.

In many cases, undifferentiated MSCs from different tissue origins and species are transplanted directly into disease models of different organ systems on different species and are reported to have positive effects but with highly varied results [12–17]. These facts suggest that MSCs possess a high level of plasticity and could contribute to the regeneration of different organs. However, there are some important parameters that are not appropriately identified and controlled for in the various studies to control these variations. One possible but undelineated explanation for the observed variation is that MSCs isolated from different tissues and species have a different lineage bias and would therefore behave differentially when implanted. MSCs are generally isolated from connective tissue by differential adhesion methods and characterised by the expression of mesenchymal markers, such as CD105 and CD73, and the nonexpression of the negative markers, such as CD45 and CD34 [18]. Although these criteria excluded contamination from other stem cell types, such as hematopoietic stem cells (HSCs), they do not declare the lineage bias of these cells appropriately. The developmental origins and lineage bias of MSCs have been a matter of debate with evidence supporting the development of MSCs from mesoderm progenitors [19], neural crest cells [20], vascular pericytes [21], and pluripotent progenitors set aside during embryonic development [22], and no concordant conclusions have been made. These data suggested that there might be multiple differentially originated and biased MSC populations from different tissues and different species.

Previous studies indicated that the extent of promiscuous gene expression and proteomic profiles of stem cells may be an indicator of its developmental bias [23]. Embryonic stem cells possess the most promiscuous expression and proteomic profiles, which is concordant with its pluripotency [24–27]. As adult stem cells, HSCs possess a relatively restricted expression profile, although the expression of differentiation genes related to their lineage progenies is widely dispersed [28]. With this rationale and with the consideration that cell surface proteins serve best for the identification of stem cells, we here present the first proteomics characterisation of cell surface proteins of rat bone marrow MSCs (rBMMSCs) and the comparison of our findings with existing datasets of the surface proteomes of mESs, hESs, and hMSCs [25, 26, 29]. These results suggest that rBMMSCs, in contrast to hMSCs, are biased towards a neural lineage and present global evidence of different lineage biases of MSCs at a proteomics level.

## 2. Materials and Methods

### 2.1. rBMMSCs Isolation and Culture

The rBMMSCs were previously harvested from Sprague-Dawley rats in our lab [30]. The cells were cultured and expanded in rBMMSC medium (*α*-MEM supplement with 15% foetal bovine serum (SiJiQing, China) and penicillin/streptomycin (Sangon, China)). Cells of passage 4–7 were used for proteomics analysis in this study.

### 2.2. Osteogenic Differentiation

For osteogenic differentiation, rBMMSCs at passage 4 were seeded at a density of 1 × 10^4^/cm^2^ in rBMMSC medium. When the cells reached 80% confluence, the medium was changed to osteogenic induction medium (rBMMSC medium supplemented with 10 mM *β*-glycerophosphate (Sigma), 50 *μ*g/mL L-ascorbic acid (Sigma), and 100 nM dexamethasone (Sigma)). The medium was changed every three days for the next 3 weeks. Then, the differentiation culture was analysed by von Kossa staining for mineral deposition.

### 2.3. Adipogenic Differentiation

For adipocyte differentiation, rBMMSCs at passage 4 were seeded at a density of 1 × 10^4^/cm^2^ in rBMMSC medium. When the cells reached 80% confluence, the medium was changed to adipocyte induction medium (rBMMSC medium supplemented with 200 *μ*M indomethacin (Sigma), 1 *μ*M dexamethasone, 0.5 mM isobutylmethylxanthine (Sigma), and 0.5 *μ*g/mL insulin (Sigma)). The medium was changed every three days for the next 2 weeks. Then, the differentiation culture was analysed by Oil red O staining.

### 2.4. Neuronal Differentiation

For neuronal differentiation, rBMMSCs and hMSCs at passage 5 were seeded in rBMMSC medium. When the cells reached subconfluence, the medium was changed to neural preinduction medium consisting of *α*-MEM supplemented with 15% foetal bovine serum and 10 ng/mL fibroblast growth factor (FGF) for 24 hours. Then, the preinduction medium was removed, and the cells were washed with phosphate buffer solution (PBS) for three times and then transferred to neural induction medium consisting of *α*-MEM supplemented with 2% dimethylsulfoxide (DMSO) and 200 *μ*M *β*-hydroxyanisole (BHA) for 19 hours.

### 2.5. Surface Protein Labelling and Protein Purification

Cell surface protein purification was performed as previously described [25]. Briefly, 5 × 10^8^ rBMMSCs at passage 4 were harvested by trypsinisation and then incubated with 1 mg/mL Sulfo-NHS-SS-Biotin (Pierce, Rockford, IL) in PBS for 30 minutes on ice. Excess biotin was quenched using 10 mM Glycine in PBS, and then the cells were washed three times with PBS. Next, the cells were homogenised in ice-cold cell lysis buffer (50 mM Tris-Cl, pH 7.4, 1% NP-40 substitute (Sigma), 150 mM NaCl, 1 mM EDTA, and 1 mM PMSF (Sigma)) using a dounce homogeniser (30 strokes). The homogenate was incubated on ice for 1 hour on a gentle vortex to extract the membrane proteins. The homogenate was then centrifuged to remove the nuclei, unbroken cells, and cell fragments. The supernatant was mixed thoroughly with streptavidin-coupled LATEX beads and incubated on ice with gentle shaking for 1 hour. The LATEX beads were precipitated by centrifugation and washed twice with a high pH buffer and once with a high salt buffer to remove the contaminant proteins. After that, the disulphide bonds linking the biotin and the purified proteins were cleaved by 100 mM DTT (Sigma) to elute the purified proteins. The labelling efficiency was monitored using FITC-streptavidin staining.

### 2.6. SDS-PAGE Separation

The purified proteins were separated by SDS-PAGE as previously described [25] and stained with Coomassie Blue R250 (Sigma). The protein lanes were dissected into eight bands and subjected to in-gel digestion.

### 2.7. Enzyme Digestion, LC-MS/MS Analysis, and Database Searching

The in-gel digestion and LC-MS/MS analysis were performed as previously described with exactly the same apparatus, LC separation conditions, and searching parameters [25].

### 2.8. RT-PCR

RT-PCR was performed as previously described [27]. Total RNA from the rBMMSCs was extracted with Trizol Reagent (Takara, Japan), retrotranscribed, and PCR amplified using the primers listed in Supplemental Table-1 in Supplementary Material available online at http://dx.doi.org/10.1155/2014/479269. Twenty amplified fragments were further confirmed by sequencing.

### 2.9. Real-Time Reverse Transcriptase-PCR

Total RNA was extracted with Trizol Reagent, and cDNA was synthesised with the ReverTra Ace qPCR RT Kit(Toyobo). Tests were performed with SYBR Green reagents (Toyobo). The primers for hMSCs were acquired from the Primer Bank (http://pga.mgh.harvard.edu/primerbank/); other primers were designed with AmplifX 1.5.2. All the primers are listed in Supplemental Table-1.

### 2.10. Antibodies

The following antibodies were used: CD73 (BD Pharmingen 551123), CD105 (Sangon DA1348), Ep-CAM (HUABIO M1111-1), CD133 (HUABIO 0804-5), PAR (HUABIO 0902-5), BMP2 (HUABIO 0806-2), LGR5 (HUABIO R1107-8), IL1RL1 (HUABIO 0902-2), CD146 (HUABIO 0804-6), CD271 (HUABIO 1003-3), CgA (HUABIO M0407-29), Noggin (HUABIO 0805-2), Alexa 488-conjugated goat anti-rabbit IgG (Invitrogen), and Alexa 555-conjugated goat anti-rabbit IgG (Invitrogen).

### 2.11. Immunofluorescence

Immunofluorescence staining was performed as previously described [25] with minor modifications. Briefly, rBMMSCs were fixed with 4% paraformaldehyde (Sigma) for 30 minutes at room temperature and then permeabilised with 0.25% Triton X-100 in PBS for 20 minutes. After blocking with 10% goat serum for 1 hour, cells were incubated with primary antibodies at 4°C overnight and then incubated with fluorophore-conjugated secondary antibodies for 30 minutes at room temperature. The nuclei were stained with Hoechst 33258 and observed under a fluorescent microscope.

Biotin-labelled rBMMSCs were fixed with 4% paraformaldehyde for 1 hour and then stained with FITC-conjugated streptavidin (Sigma) for 30 minutes to monitor the surface labelling.

### 2.12. Western Blotting

Western blotting analysis was performed as previously described [27].

### 2.13. Bioinformatics Analysis

The subcellular localisation of the proteins was annotated according to the Swissprot annotation, the SOSUI prediction software, and the literature [31]. Proteins containing transmembrane domains, secreted proteins, and proteins annotated as cell surface proteins by either Swissprot or the existing literature were all considered cell surface proteins. Surface proteomics data of the mESs and hESs were from our previous publication, and the data of the hMSCs were downloaded from the PLoS ONE website [29]. Gene ontology (GO) and tissue specificity analysis was performed using the DAVID software and database [32, 33].

## 3. Results

### 3.1. Isolation and Characterisation of rBMMSCs

**Figure 1:**
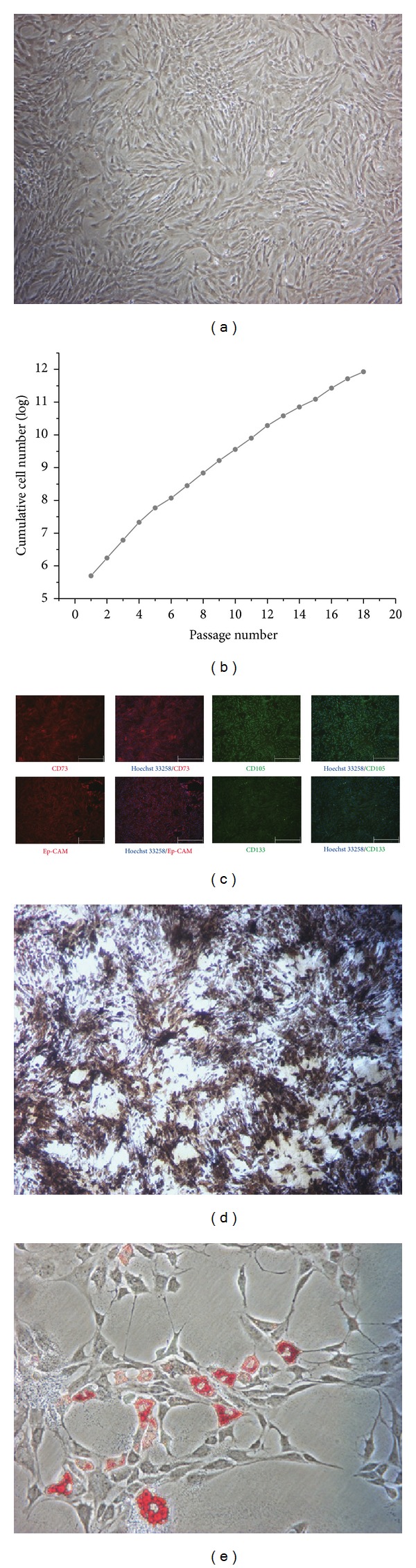
Characterisation of rBMMSCs. (a) The morphology of rBMMSCs under a phase contrast microscope. The MSCs displayed a spindle-shaped morphology (10x). (b) The growth curve of rBMMSCs through 18 passages (*n* = 3). (c) The expression of cell surface markers on rBMMSCs (the bars represent 500 *μ*m). (d) von Kossa staining of rBMMSCs induced with osteogenic induction medium for 3 weeks showed accumulation of mineral depositions (*n* = 3). (e) Oil red O staining of rBMMSCs induced with adipogenic culture medium for 2 weeks showed lipid accumulation in cells (*n* = 3).

The rBMMSCs showed the typical spindle like morphology in culture (Figure 1(a)) and possessed sustained proliferation capability for more than eighteen generations, which is consistent with typical MSCs (Figure 1(b)). The rBMMSCs expressed the MSC markers CD73 and CD105 (Figure 1(c)) and did not express the hematopoietic markers (described in our previous publication) [30]. Moreover, the rBMMSCs also expressed other somatic stem cell markers, including Ep-CAM and CD133 (Figure 1(c)). The differentiation potential of the rBMMSCs was tested by induced differentiation into osteoblast and adipocyte lineages. The rBMMSCs possess the potential to differentiate into both lineages as shown by von Kossa and Oil red O staining, respectively (Figures 1(d) and 1(e)). These data indicated that the rBMMSCs possess the typical characteristics and differentiation potentials of MSCs.

### 3.2. Surface Proteomics Analysis of rBMMSCs

**Figure 2:**
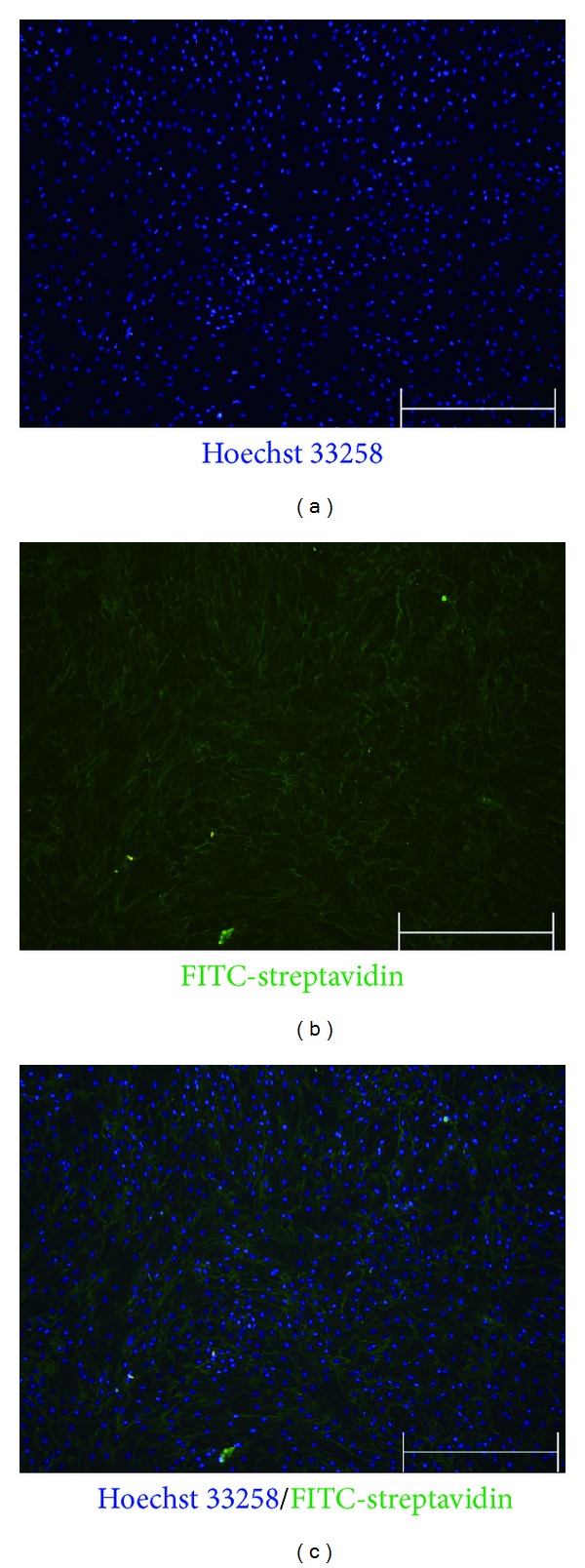
Labelling of rBMMSCs surface proteins: staining with Hoechst 33258/FITC-streptavidin showed a strong preference toward cell surface proteins (the bars represent 500 *μ*m).

As we and others have previously demonstrated, membrane impermeable biotin labelling is an efficient method to purify surface proteins for proteomics analysis on cultured stem cells [25, 26, 29]. Therefore, we used this method to analyse the cell surface proteome of rBMMSCs. As labelling selectivity and efficiency were both critical parameters that affect the analysis, we first examined the labelling of rBMMSCs cell surface proteins by streptavidin-FITC staining. As shown in Figure 2, a strong and clear labelling of the cell membrane was presented on most cells, indicating that the cell surface proteins were selectively labelled on most cells as we have performed previously. The cells were then lysed, and surface proteins were purified with streptavidin-conjugated LATEX beads and subjected to LC-MS/MS analysis. The analysis resulted in the identification of 2637 proteins among which 674 were categorised as transmembrane proteins, lipid anchored proteins, or secreted proteins. We selected these proteins as putative surface proteins for further analysis (Supplemental Table-2). The efficiency of surface protein identification was similar to that shown in our previous report on ES cells and sperm, suggesting a similar purification and identification efficiency [25, 26]. To confirm the true identification rate of the analysis, we randomly selected 65 cell surface proteins and examined their expression in rBMMSCs by RT-PCR. Of the 65 proteins, 63 were confirmed to be expressed in rBMMSCs, indicating a low rate of false identification. Moreover, the expressions of several surface proteins were also confirmed at the protein level (described later).

### 3.3. Functional Characteristics of rBMMSCs

**Figure 3:**
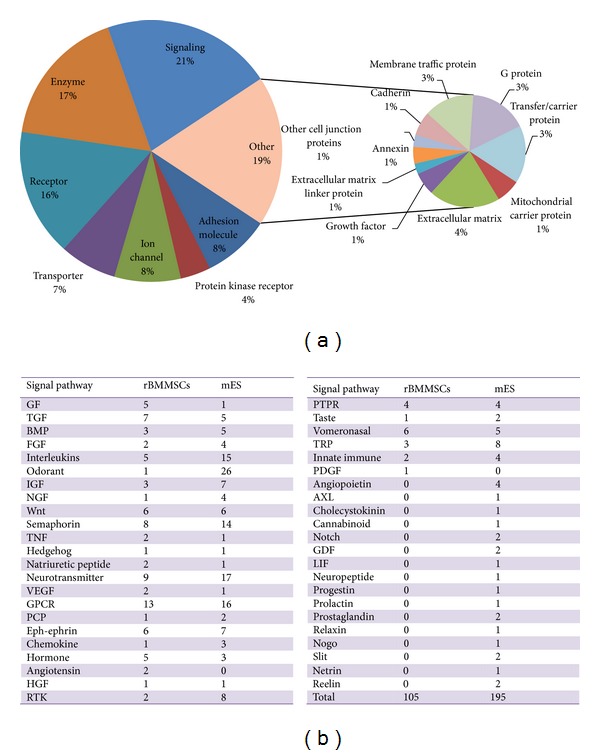
Gene ontology surveys and signal pathways of rBMMSCs. (a) GO surveys showed the enriched surface proteins associated with transportation or ion channel activity. (b) Signalling pathways identified in this study and the comparison with mES signalling pathways identified in our previous study.

To reveal the functional characteristics of the surface proteome of rBMMSCs, we performed a gene ontology (GO) survey on molecular function (MF) with the DAVID software. As shown in Figure 3(a), most of the enriched MF categories in rBMMSC surface proteins were associated with transportation or ion channel activity. We compared the GO distribution of rBMMSCs surface proteins with our previous results on ES cells. The results showed that rBMMSCs were less enriched for surface proteins serving differentiation-regulating functions, such as receptor activity and cell adhesion [25, 26]. Therefore, in contrast to ES cells, more rBMMSC surface proteins serve physiological functions, such as material transportation, indicating that MSCs occupy a more differentiated state and possess less plasticity than ES cells. However, it is noteworthy that the gated ion channel function, which is generally performed by neural receptors, is highly enriched in rBMMSCs and presented a first hint of the neural lineage bias of these cells.

### 3.4. Signal Transducers in rBMMSCs

Signal transducers play critical roles in the identity maintenance and plasticity of stem cells. To this end, we analysed the signal ligands and receptors on rBMMSCs to reveal the signalling pathways that function in these cells. As shown in Figure 3(b), signal receptors and ligands from 30 categories of signalling pathways were identified in rBMMSCs. The signal pathways include growth factor pathways, including those associated with epidermal growth factor (EGF) [34, 35], transforming growth factor (TGF) [35, 36], fibroblast growth factor (FGF) [35, 36], hepatocyte growth factor (HGF) [37], and platelet derived growth factor (PDGF) [35, 36], suggesting that the growth and survival of rBMMSCs are regulated by complex growth factor corporations. Many developmental regulation pathways, such as those involving Wnt [38], bone morphogenetic protein(BMP) [39], sonic hedgehog [39], planar cell polarity (PCP) [40], and eph-ephrin [41], were also identified in these cells. These pathways might be responsible for the differentiation plasticity of the rBMMSCs. Also noteworthy, eight receptors and ligands from the semaphorin signalling pathways, which have been characterised intensely in directing neural development, and nine receptors from neural transmitter pathways, including acetylcholine receptors, gamma-aminobutyric acid receptors, and glutamate receptors, which mediate the physiological functions of neural cells, were identified. Moreover, receptors and ligands that function in some specific branches of neural lineage cells, such as the olfactory receptors, taste receptors, vomeronasal receptors, and TRP channels, were also identified in BMMSCs. This further suggested the neural lineage bias of these cells. We also compared the signal transduction pattern of rBMMSCs with our previous data on mouse ES cells (mESs). The results showed that rBMMSCs expressed fewer signal receptors and ligands than mES cells. Additionally, rBMMSCs expressed fewer categories of signalling pathways than mES cells. These results suggest that, as more committed adult stem cells, rBMMSCs depend on a less complex signalling network for their sustenance and plasticity.

**Figure 4:**
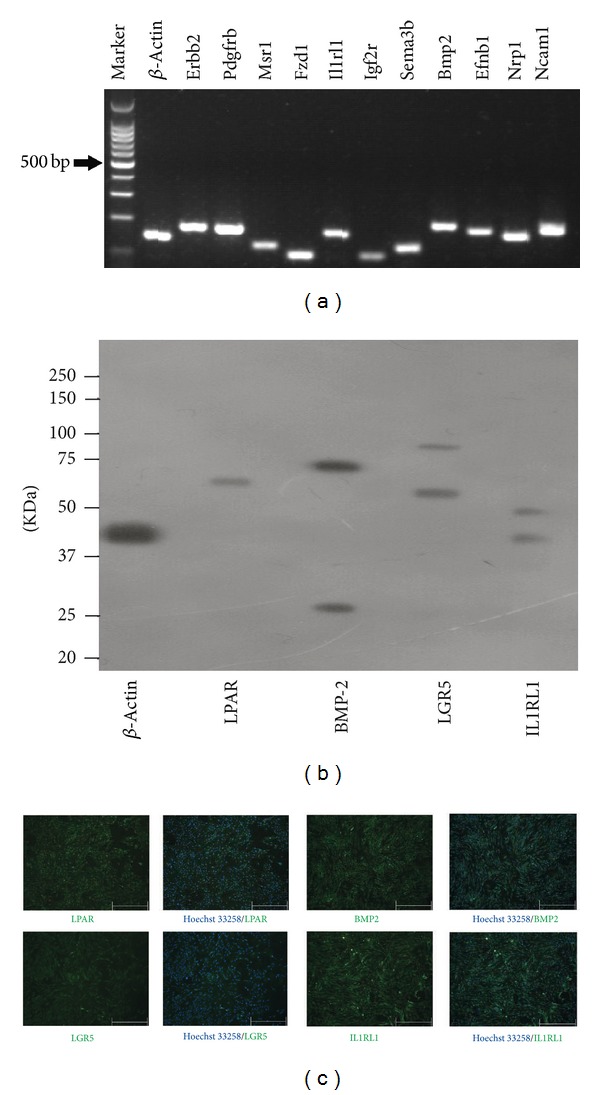
Confirmation of the signal proteins identified: (a) RT-PCR confirmed the expression of signalling receptors and ligands on rBMMSCs (*n* = 2). (b) Western blotting confirmed the expression of LPAR, BMP-2, LGR5, and IL1RL1 on rBMMSCs (*n* = 2). (c) The expressions of LPAR, BMP-2, LGR5, and IL1RL1 were confirmed by immunofluorescence (the bars represent 500 *μ*m).

In addition to proteomics analysis, we also confirmed the identified signalling proteins at mRNA and protein levels. As shown in Figure 4(a), the mRNA levels of eleven signalling receptors and ligands from ten different signalling pathways were presented on rBMMSCs. Western blotting and immunofluorescence analysis confirmed that rBMMSCs expressed LPAR, BMP2, LGR5, and IL1RL1 (Figures 4(b) and 4(c)), suggesting a functional role of these signalling pathways in rBMMSCs.

### 3.5. Tissue Specificity of rBMMSCs Cell Surface Proteins

**Figure 5:**
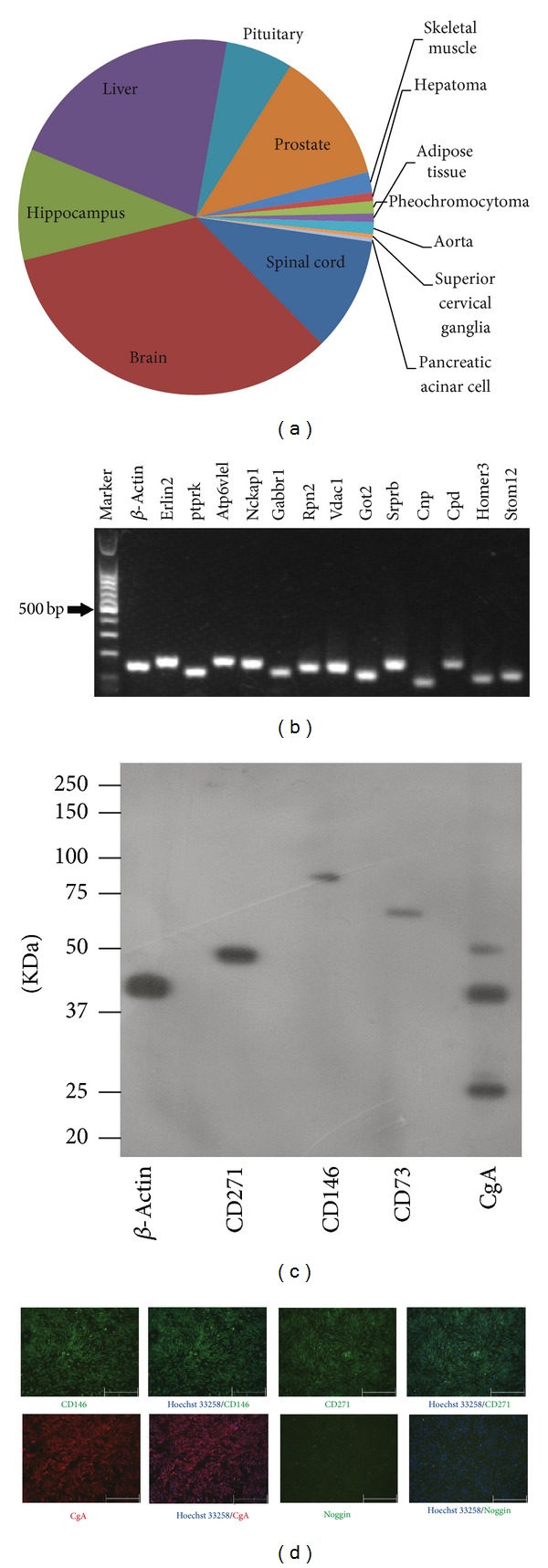
Tissue specificity analysis and confirmation of rBMMSCs surface proteins. (a) Tissue specificity distribution of cell surface proteins. (b) RT-PCR confirmed the expression of neural-biased lineage markers on rBMMSCs (*n* = 2). (c) Western blotting confirmed the expression of neural biased lineage markers: CD271, CD146, CD73, and CgA (*n* = 2). (d) Immunofluorescence further confirmed the expression of CD146, CD271, CgA, and Noggin (the bars represent 500 *μ*m).

To reveal the possible lineage bias of rBMMSCs, we analysed the tissue specificity distribution of rBMMSCs surface proteins using DAVID software (Figure 5(a)). Interestingly, of the thirteen tissue types, nine were of neural or neural endocrine lineages (brain, hippocampus, spinal cord, pituitary, prostate, superior cervical ganglia, pancreatic acinar cells, and pheochromocytoma) or could be traced to the neural crest as a developmental origin (adipose tissue and the aorta). The nonneural tissue types enriched in the analysis were liver, hepatoma, and skeletal muscle. As liver tissue expressed a large variety of proteins and these proteins were enriched during all the analyses of the cell surface proteome on tissue specificity [25, 26], it is possible to rationalise presence in the analysis. The same rationale can also be applied to hepatoma. A detailed analysis of proteins in skeletal muscle revealed that the proteins that function in neural-muscular junctions contribute significantly, providing further evidence that rBMMSCs stem from a neural lineage bias. To confirm this hypothesis, we examined and demonstrated the expression of neural biased lineage markers on rBMMSCs at both mRNA and protein levels (Figures 5(b) and 5(c)). These results strongly suggest that rBMMSCs possess neural biased lineage properties.

**Figure 6:**
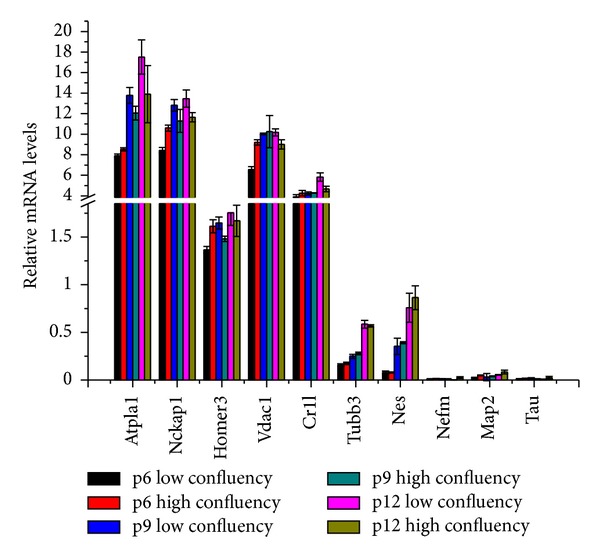
Expression levels of neural and neural-endocrine genes at different passage and confluency status. p6, p9, and p12 represent passage 6, passage 9, passage 12, respectively (*n* = 3).

We used newly seeded rBMMSCs for the following analysis. The results suggest that rBMMSCs have neural biased lineage properties. To investigate whether these neural biased lineage properties of rBMMSCs change with number of passages or confluency status, samples of passage 6, 9, and 12 at confluence status of 70% or 90% were used for the analysis. Several neural- and neural-endocrine-associated genes were randomly chosen for the assessment. The expression of the relative mRNA levels exhibits some fluctuation between different confluency statuses (Figure 6) but presents an overall slightly upward trend as the passage number increase. The results indicate that the neural and neural-endocrine bias trend will be maintained as the passage number increases.

**Figure 7:**
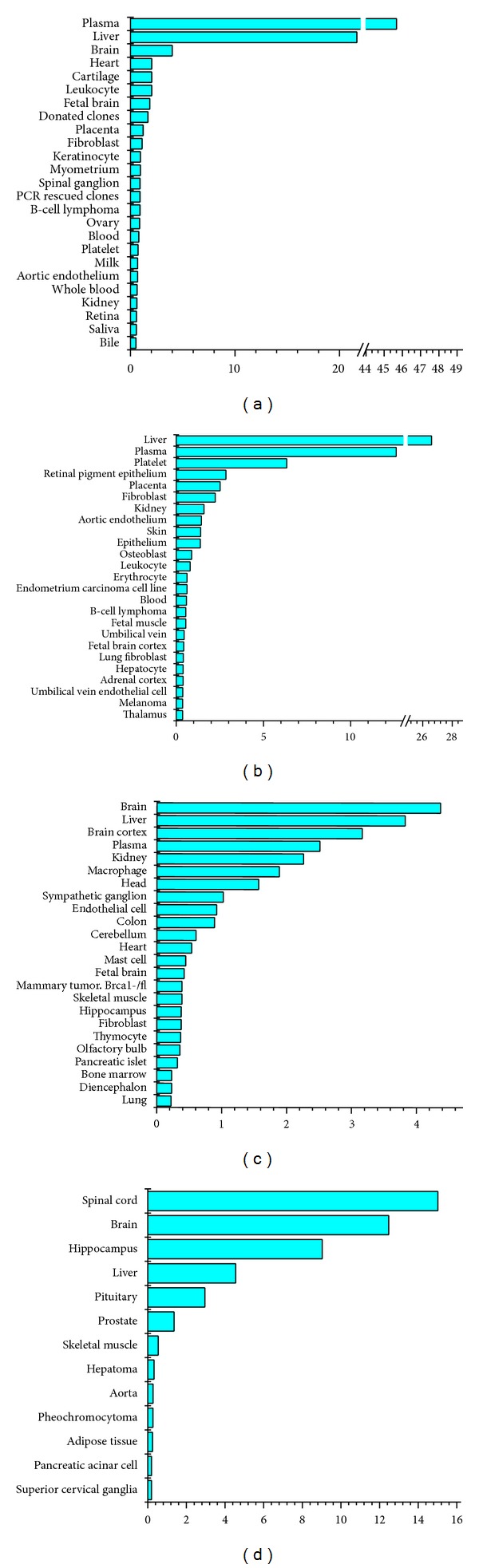
Comparison of cell surface tissue specificity. (a) Tissue specificity profile of hESs. (b) Tissue specificity profile of hMSCs. (c) Tissue specificity profile of mESs. (d) Tissue specificity profile of rBMMSCs.

To gain more insight into the relationship between the tissue specificity profile of cell surface proteins and the plasticity level of stem cells, we compared the tissue specificity enrichment profile of rBMMSCs surface proteins with the profiles of mouse and human pluripotent ES cells that we previously reported. As shown in Figure 7, although ES cells were also enriched for neural lineage specific surface proteins to some extent, which could be explained by their default differentiation route to neural lineages, they also expressed a large variety of surface proteins of tissue types of all three germ layers. In contrast, most of the enriched tissue types were of neural or neural-endocrine lineages, including spinal cord and hippocampus, in rBMMSCs. These data indicated that, in contrast to the pluripotent ES cells, rBMMSCs possessed a tissue specific surface proteome profile that was more restricted to neural lineages further suggesting their neural lineage bias.

There is currently no consensus view concerning the consistent developmental identity of MSCs from different species and tissue types. We sought to examine the surface proteomics data of rBMMSCs from a global viewpoint by comparing our data with the surface proteomics data of human mesenchymal stromal cells published by Niehage et al. [29]. To our surprise, the tissue specificity of surface proteins was obviously distinct between these two cell types. While rBMMSCs were enriched for neural or neural-endocrine lineage surface proteins, hMSCs possessed a more wide-spread expression profile of tissue specific surface proteins that covered many cell lineages of the three germ layers. These results demonstrated that the hMSCs expressed significantly different tissue specific surface proteins than those expressed by rBMMSCs, suggesting that MSCs from different species might occupy different developmental stages and possess different lineage biases.

### 3.6. rBMMSCs Are More Sensitive to Neural Induction Compared with hMSCs

**Figure 8:**
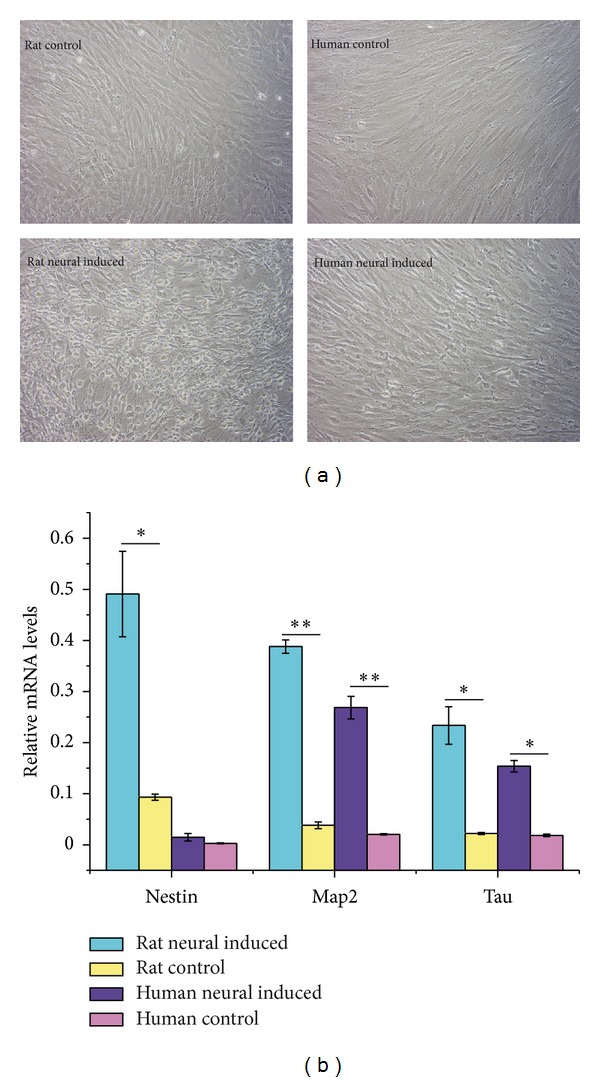
Neural differentiation potential. (a) The morphology of rBMMSCs and hMSCs under regular culture conditions and neural induction for 2.5 hours. (b) The relative mRNA levels of neural genes in rBMMSCs and hMSCs (*n* = 3, *P < 0.05, **P < 0.01) after 19 hours of neural induction.

For the analysis of neural differentiation, we used DMSO/BHA, the chemical compound widely used for neural induction in vitro. After neural induction for 2.5 hours, a large majority of rBMMSCs adopted a neuron-like morphology, but of hMSCs, only a small percentage of cells adopted a neuron-like morphology (Figure 8). Furthermore, after neural induction for 19 hours, we tested the expression of neural associated genes. Both in rBMMSCs and hMSCs, the neural-induced cells have a higher gene expression than the control groups, but in rBMMSCs, there is a higher relative mRNA expression level of these genes than in hMSCs. Considering the rapid morphology change and neural-associated gene expression, these results serve as an indicator that rBMMSCs seem more sensitive to chemical induction than hMSCs. This may also suggest that rBMMSCs are easy to differentiate into neural cells.

## 4. Discussion

Although widely considered as a promising cell source for regenerative medicine, the molecular characterisation of MSCs was relatively vague. A panel of positive and negative surface markers has been routinely used to identify MSCs, but there are still strong variations in marker expression through MSCs originating from different tissues and species [42, 43]. Consistent with this, MSCs with different origins showed highly variable potentials during in vitro differentiation and transplantation [44, 45]. Globally characterising the surface proteomes of MSCs and comparing the surface proteomic characteristics of MSCs from different origins would shed deep insight into the factors that determine the variations and the selective and quality control criteria in MSCs applications. Moreover, as the extent of gene expression promiscuity had been proposed to reflect the differentiation plasticity of stem cells [23–27], comparing the surface proteomic characterisation between MSCs of different origins and between pluripotent stem cell types would highlight the implications of understanding the varied differentiation bias of MSCs. Here, we presented the surface proteomic characterisation of rBMMSCs and compared the data with the surface proteomics datasets on mESs, hESs, and hMSCs published previously [25, 26, 29]. The very same methodology used to acquire these data ensured the rationale of the comparison. The results revealed very distinct surface proteomic characterisation of rBMMSCs compared to the three characterised stem cell types and presented implications on the molecular properties of MSCs.

### 4.1. Relatively Restricted and Neural Biased Signalling Molecular Expression in rBMMSCs

As signalling molecules performed critical roles in stem cell sustenance and differentiation, we analysed the signalling molecules identified in rBMMSCs and compared them with our previously published data on mES cells [25]. The results revealed that compared with mES cells, which are pluripotent, rBMMSCs possessed a relatively restricted signalling molecular repertoire of signalling receptors and ligands. Together with the previous evidence hinting that hematopoietic cells gradually restrict their signalling capacity with differentiation [28], these results indicate that the restriction of signalling capacity plays roles in the restriction of differentiation plasticity during differentiation. Another intriguing fact is that rBMMSCs expressed a relatively large variety of signalling molecules of neural lineages compared to those expressed by mES cells, indicating a neuronal biased state of these cells.

### 4.2. Neural Biased Tissue Specific Surface Proteins Expression in rBMMSCs

There is currently no consensus on the understanding of the lineage origin and developmental bias of MSCs. Candidates, including mesoderm progenitors [19] and neural crest progenitors [20], have been proposed. Although it had been shown that undifferentiated MSCs expressed some neural markers such as nestin and *β*-III tubulin and possessed intrinsic differentiation capacity to neural lineage [46–49], no proteomics data are available currently that provide support for or against neural lineage bias of these cells. Here, our data showed that rBMMSCs expressed a large variety of neural and neural-endocrine tissue specific proteins and expressed little surface proteins specific to other tissue types except for the liver, which is an organ with a relatively promiscuous gene expression profile. Compared to ES cells, which are pluripotent, the expression profile of tissue specific surface proteins in rBMMSCs is highly restricted to neural lineages indicating that these cells possess a biased potential to neural lineages than pluripotent stem cells [25, 26]. More interestingly, comparing the surface proteomics data from rBMMSCs with the published data on hMSCs revealed a distinct profile [29]. While rBMMSCs showed a neural biased surface protein profile, hMSCs showed a more wide-spread and balanced profile. These data indicated that while rBMMSCs were neural biased, hMSCs were still in a multipotent state. This evidence would help to explain some of the variability of differentiation potential between MSCs from these two species and indicate the importance to select the appropriate type of MSCs for certain applications.

### 4.3. More Sensitive to Chemical Induced Neural Differentiation

The process of neural differentiation in MSCs is currently heavily debated. Many challenges are present that question the authenticity of neural differentiation in MSCs [50–52]. Chemical induction for neural differentiation in MSCs has some limitations, and many factors need to be further examined. It has been proposed that neuronal differentiation of stem cells is regulated by specific and temporally precise genetic events [53–55]. Although the precise mechanism of chemically induced neural differentiation has not been clearly shown, it can be agreed that, during the chemically induced neural differentiation, there are some temporally regulated genetic events. Research found that the mRNA levels of several neural genes changed rapidly during neural induction [56]. This temporal change in expression of mRNA may account for apparent expression change of neural associated proteins. Considering the change in morphology upon chemically induced neural induction, the increase in neural-associated protein levels and the neural gene expression change, chemically induced neural differentiation, to some extent, can be used as an indicator for the neural differentiation potential of MSCs. Neural induced rBMMSCs have a higher rate of neural-like morphology change and a higher relative expression of neural-associated genes compared to hMSCs. These results suggested that rBMMSCs may more easily differentiate into neurons than hMSCs.

In conclusion, we have characterised the surface proteome profile of rBMMSCs and revealed that from the proteomics viewpoint rBMMSCs possessed a more restricted profile than pluripotent ES cells and multipotent hMSCs. The tissue specificity of rBMMSCs is highly restricted to a neural lineage, which provided evidence that these cells may originate from a neural lineage bias. These results provided some explanation into the varied differentiation bias of MSCs from different species and tissue types, and future directions will shed light on the induced differentiation and therapeutic application of MSCs.

## Supplementary Material

The primers for human and the primers for rat including Nes, Nefm, Tubb3, Map2, Tau, Atp1a1, Nckap1, Homer3, Vdac1 and Cr1l were used for real-time RT-PCR. Primers including Atp1a1, Nckap1, Homer3, Vdacc1 and Cr1l which were also identified in proteomics analysis and other 60 primers for rat were used for RT-PCR identification, of the 65 identified proteins, 63 were confirmed to be expressed in rBMMSCs.
