# Supplementary material for: Cell Surface Proteomics Analysis Indicates a Neural Lineage Bias of Rat Bone Marrow Mesenchymal Stromal Cells
Source: Biomed Res Int. 2014 Jan 16;2014:479269. doi: 10.1155/2014/479269 (PMC3914342; doi:10.1155/2014/479269)
Supplement: Supplementary file 1 — The primers for human and the primers for rat including Nes, Nefm, Tubb3, Map2, Tau, Atp1a1, Nckap1, Homer3, Vdac1 and Cr1l were used for real-time RT-PCR. Primers including Atp1a1, Nckap1, Homer3, Vdacc1 and Cr1l which were also identified in proteomics analysis and other 60 primers for rat were used for RT-PCR identification, of the 65 identified proteins, 63 were confirmed to be expressed in rBMMSCs. [file 479269.f1.pdf]

Supplemental Table-1: Primers used in this study

Primers	Primers for rat<br>Forward(5'-3')	Reverse(5'-3')
	Primers for rat	
Erlin-2	CAGTTCTGCAGCGTCCATACACTT	AACTCATAGTTCCTGCGGATTGCC
Gabbr1	TTCACCAGCCTTGTCAAACCGA	TGGTCTGTTGGATGGTAGCGATCT
Ptprk	TGCGGACGCCAAAGACTTTGAA	GGCCTCGGAAGTAATGGTAGCAAATG
P2rx2	TGGACATGCAAGGCAATGGGAT	ACTTGGGGTAGTGGATGCTGTTCT
Tpbg	TGCATCTTTCCGCAACCTGACA	GTAGCAGTCACAAACCCAGGGATT
Atp6v1e1	GCAAGACTCAAAGTCCTCAGAGCAAG	GCACAATCATTCGAGGTTCCAACAGC
Fxyd5	AAGACCCAGCAACTGACCGAAA	AGAAGGGGCTGTTCTCATTGGGAT
Aplnr	TTTGCCACTGTGGGCCACTTAT	ACAATGGCCAGGTATCGGTCAAAG
Rpn2	TCCCAGAATCGCTATCACGTACCA	AAGCCACGGACTTAGCATGTTCCA
Atp1a1	ATGAGCATCAACGCAGAGGATGTC	TGGGGTTCTGATTCACCAGTGAGT
Rell1	TGGTGTCCTCATCTGCCACTTACT	TATAGTGGACAATCTGCCCAACGGT
RT1-EC2	ATTACCGAGTGAGCCTGAGGAA	AAGTCTGCCACAGCCCATGTTT
Alcam	ATGGCATCTAAAGGGTCCCCTTCT	GTTCTGAGGTACATCCAGTCTGCAAG
Crl1	CTGTTCTTGTCGCCATCTACTTTGGG	CCACACTGAGTTTACTTCGCAGGT
Atp1b1	ATTGTGGCAGTATGCCCAGTGAAC	TCCCCTCTTTGTAGCCGTAGGATT
Fat2	AATCTCGGAGGGCAATGGATTTGG	AGGGAATCTACTTCAGCTCCTGAACC
Nckap1	GTGCTTGGAGTTGCCTTCTCAGT	TTCCGAGCTTGTTTGGCCATCT
Vdac1	CCACATATGCTGATCTTGGCAAGTCC	GGTTTCCAGACTGCCGTTCACTTT
Got2	CCATGTTGAAATGGGACCTCCAGA	GGAGCACGTAAGGCTTTCCGTTAT
Srprb	CAGGGGTAACAGCCTGACTTTGATTG	CTGTCGATGAGGACCTGGTACAGAA
Atp5a1	CGGCTTAAAGGGTATGTCCCTGAACT	ACCCTTCCCATCAATGGCATTTCC
Cnp	GAGATCTTACGGCAAGGCCTTCAA	ACAACTGCAGCTCCTGCTCATT
Cpd	ATCACAGCATCTGCTCGAGGGTAT	TTAGAAGTTGGGTCGCTGGTGTCATC
Gabbr1	CACCCGAAGCATTTCCAACATGAC	AAAGCCAAGGCCCAGATAGCATCA
Ncam1	ATACACCATCTTCCCCATCCATCGAC	GTGACAATCCCTTCCATGTTGGCT
L1cam	GCACCATCTGGCTGTGAAGACTAA	TGAAGCAGAGGATGAGCAGGATGA
Cdh10	CCATCCATCTGCGAGTTCTTGAATCC	GCGGTAATCCACTTCGGCATTT
Cdh23	CGTGATCACAGTCAAAGGCTTGGT	TGAAGTCAAAGCGTGGGCTGTT
Cdh17	CAGCATATTTCCACCTGGTCCTCA	TCTCTGCCAAGGGCAAACGTAAAC
Cdh1	TAAAGACCAGGTGACCACGTTGGA	CCAAGGATGGCGGGAACTTGTAAT
Itga5	TCAACCTTAGCTTCTGCCTCAACG	CATTCTGGATAAGCAGGGTCTGGGTA
RHAMM	AACAGTTGAAGCTTGCCCTGGATG	TTCCGCAGTTGACTTCGTTTCCTC
Itgb1	CGATAGGTCCAACGGCTTAATCTGTG	CTTGGAACTTGGGATCTGTGCACT
Marco	AGTCCCAGGTCTTGTAGGCAGAAA	CGATCCGGACAACCAGGTTGAAAT
Msr1	TCCTAAAGGTGATCAGGGACCAGTTG	GATATTCCTGCACGCCCCTTTTCT
Tlr10	TGCTGGTTATCCTGGTTCTGGGTT	GACAGAATCATGTGCAGCGTAGGAGA
Tlr12	ACCCTTAGCCTGCTAGATACCCAA	GACAGTGTCCAATGCTTCAAGCCT
Trpm2	CTGAGATCACCATCTCCCTGATCCA	GCAGAATGGCAACATCCACATCCT
Trpm8	GCTAATGAGTATGAGACCCGAGCAGT	CAATGAAATGCTGGTCCGTAGCCT
Ryk	TTAACCTGCTAGTGCCCAGTGAGA	TACTTTGCCGGTACACGAAAGCTC

Fshr	TGACATCCATACCAAGAGCCAGTACC	AGCTGCACCTTGCATTCCAGTT
Efnb1	CCAACTACATGGGCCTGGAATTCA	TGGTAGTCAACTGCTCAGGCGTTA
Celsr3	CGTACGAGTGCACATAACTGTGCT	ACCAAACCATTGGCATCCTTGTCC
Grm7	GAGATGACCTGTCAGCATTGTCCCTA	CCAACATTGCCAGGAAGACAGGAA
Grik4	AACTGAACCGGAGCCAGGAAATTG	CGAAACCCATTCCTGGTGAACTGT
Chrm5	TTCCCAGTGTCCAAAGACCCTTCA	ACAGAAGGTGGAAACCAGGACCAT
Npr1	ATATGCGGGATGTGCAGAATGAGC	TGTCATTGGTGAGCGAGTACCGAA
Pdgfrb	GTGAATGACCACGGCGATGAGAAA	CAAGGTACGGTTGTCCTTGAACCA
Pdgfb	CGGTGCAGGTGAGAAAGATCGAAAT	AGTTTGAGGTGTCTTGGCTCGATG
Nrp1	GTTGGGCATGGTGTCTGGACTTAT	CAGGTCCACTTGGAGCCATTCATT
Sema3b	AACATCAGCAAGCGAGCCAAGA	GTTGTAGGCATGCAACAGCTTCAC
Sfrp4	TTGCGATGAGCTGCCTGTCTATG	CGATCAGGGCTCAGATGTTTACAGTC
Fzd1	CTCATGAACAAGTTCGGCTTCCAGT	TGCCTTTGTCGGAAGTGTTCTG
Igflr1	AAAGTCCGTTCCCATCCTTGTTCC	GATGTCTTCCCTCTGTTTCCACTTCC
Igf2r	AATTGGGAAGCAGTGGATGGCA	ACCTTCAGGACAGTTCTTGGCCTTTC
Il1rl1	GAGTTTGCCTATGAGCAGGAGATCG	CCACTTGATGGTCCCCTGCATTTT
Il23a	TTTGCAAAGGATCCGCCAAGGT	ATCTGTTGAGTCTCCCAGTGGT
Fgf9	CGCAGTCACGGACTTGGATCATTT	ACCAGGCCCACTGCTATACTGATAA
Bmp2	GACCAGACTATTGGACACCAGGTT	CTTGGTGCAAAGACCTGCTAATCCTC
Fstl4	AGTGCTGACCTGTGCTATTCGT	GGTGTAGTTGCCCATGTGGATTGT
Mstn	TCCTCAGTAAACTCCGCCTGGAAA	CCGTGGTAGCGTGATAATCGTCAT
Erbb2	GCTCTGCTACCAGGACATGGTTTT	GGTACAGATGGTGCCAGTCAAGAT
Stoml2	GTGAGTCCCTGAACGCCAACATT	CCTGCATCTGCATGGACTCTTTGA
Sept3	GGGAGTGACAAGGAGTACCAAGTGAA	TTGAGCCTCTTGGCCCTGTAAGTT
Homer3	CAAGCATGCACTTACCGTGTCCTA	TGAAGGTCATGTTGGGAGTGACTGT
CD31	TGTCTACTCAGTCATGGCCTTGGT	CGGCCTGGGAAACAGTTCTGTTAT
CD45	GCTCAAACTTCTGGCCTTCAGCTT	GTGAGGCATCAGCGTGGATGAAAA
CD44	CCCTGAGAAAGGGGTTTGAAACATGC	TGTGTCATAGTGGGAGGTGTTGGA
Nes	AGGTGGGTGCTCTAAAGGTT	AGGATCTCACCTCCCTTGCT
Nefm	AGCCAAGGAATCACCCAAGGAAGA	TCCAGGCTCACCTTTACCGACTTA
Tubb3	TGCATCGACAATGAAGCCCTCTAC	CATTGAGCTGACCAGGGAATCGAA
Map2	GCAGAAATTGCCTTCCTCATTCGC	TCCTTCCATGTCTTGGGGATCCTT
Tau	ACAGACCATGGAGCAGAAATCGTG	ACAAACCCTGCTTGGCCAAAGA
Actb	GTGACGTTGACATCCGTAAAGA	GCCGGACTCATCGTACTCC
	**Primers for human**	
Nes	CTGCTACCCTTGAGACACCTG	GGGCTCTGATCTCTGCATCTAC
Map2	CTCAGCACCGCTAACAGAGG	CATTGGCGCTTCGGACAAG
Tau	CCAAGTGTGGCTCATTAGGCA	CCAATCTTCGACTGGACTCTGT
ACTB	CATGTACGTTGCTATCCAGGC	CTCCTTAATGTCACGCACGAT

Supplemental Table-2:Cell surface proteins identified in this study

NCBI Protein Gi	Protein Name
158138551	alpha-2-macroglobulin
333033773	Alpha-fetoprotein
6978501	Annexin A1
6978509	Adenomatous polyposis coli protein
6978527	Aquaporin-1
6978539	Beta-arrestin-2
6978543	Sodium/potassium-transporting ATPase subunit alpha-1
6978547	Sodium/potassium-transporting ATPase subunit alpha-3
148747253	Sodium/potassium-transporting ATPase subunit beta-1
6978553	Sodium/potassium-transporting ATPase subunit beta-3
6978555	Sarcoplasmic/endoplasmic reticulum calcium ATPase 3
158138532	Copper-transporting ATPase 2
6978595	Calcium/calmodulin-dependent protein kinase type II subunit delta
6978647	Secretogranin-1
6978667	Chymase
6978695	Ceruloplasmin
6978697	Carboxypeptidase B
6978699	Carboxypeptidase D
6978773	Dipeptidyl peptidase 4
6978785	Proheparin-binding EGF-like growth factor
6978797	Pro-epidermal growth factor
158186649	Alpha-enolase
6978857	Follistatin
6978867	Gamma-aminobutyric acid receptor subunit beta-1
6980972	Aspartate aminotransferase, mitochondrial
6980990	Gastrin-releasing peptide receptor
6981068	Intercellular adhesion molecule 1
6981108	Integrin beta-4
6981110	Inositol 1,4,5-trisphosphate receptor type 3
6981142	Laminin subunit beta-2
6981164	Hormone-sensitive lipase
6981214	Matrilysin
6981280	Atrial natriuretic peptide receptor 1
6981354	Multidrug resistance protein 3
6981420	Anionic trypsin-1
6981468	Peripherin-2
6981590	Interleukin-1 receptor-like 1
148368981	ATP-binding cassette sub-family C member 8
7549773	Kininogen-1
8392842	Lysosomal acid phosphatase
8392852	Adenylate cyclase type 8

291327518	Vasopressin-neurophysin 2-copeptin
8392990	Bone morphogenetic protein 2
8393030	Voltage-dependent L-type calcium channel subunit alpha-1D
8393087	Cell adhesion molecule-related/down-regulated by oncogenes
8393123	Muscarinic acetylcholine receptor M5
8393138	Chloride channel protein 2
158517923	Atrophin-1
8393399	Gamma-aminobutyric acid receptor subunit rho-1
8393487	Metabotropic glutamate receptor 1
8393576	3-keto-steroid reductase
158303324	Integrin beta-1 CD29
8393649	ATP-sensitive inward rectifier potassium channel 1
8393820	Neural cell adhesion molecule L1
8393929	cGMP-inhibited 3',5'-cyclic phosphodiesterase B
8394121	Peroxisome biogenesis factor 2
8394142	Ras-related protein Rab-27A
126723574	Adenylate cyclase type 4
9506403	ADP-ribosylation factor-like protein 4A
9506421	Beta-secretase 1
9506595	Fibroblast growth factor 18
186972114	Fibronectin
9506883	Membrane cofactor protein
9506901	Multiple PDZ domain protein
9506903	Beta-microseminoprotein
9506907	Growth/differentiation factor 8
9845234	Annexin A2
9845261	Galectin-1
9910120	Disintegrin and metalloproteinase domain-containing protein 2
9910340	Arf-GAP with dual PH domain-containing protein 2
10048483	Protein piccolo
10242377	Glutamate receptor, ionotropic kainate 4
158186718	Frizzled-1
11072106	Nucleobindin-2
11177892	Potassium channel subfamily T member 1
11276093	Vascular endothelial growth factor receptor 1
11559947	Peripheral plasma membrane protein CASK
11596861	Guanylate cyclase activator 2B
11612653	FXYD domain-containing ion transport regulator 5
12018314	Phosphorylase b kinase regulatory subunit alpha, skeletal muscle isoform
12083643	Exocyst complex component 7
158636004	Flotillin-1
12621132	Protocadherin Fat 2
12711694	Cytoplasmic dynein 2 heavy chain 1
13027426	PRA1 family protein 3

13162326	Bile acyl-CoA synthetase
13242253	Bifunctional heparan sulfate N-deacetylase/N-sulfotransferase 1
13540622	Glypican-1
13540640	Solute carrier organic anion transporter family member 1A3
13540689	Moesin
13540699	Neuropilin-2
256221435	Plectin
13569846	Alpha-parvin
13591902	Alpha-actinin-1
13591914	Aminopeptidase N
13591932	Chondroitin sulfate proteoglycan 4
13591955	Guanine nucleotide-binding protein G(i) subunit alpha-2
13591963	Metabotropic glutamate receptor 7
146262019	72 kDa type IV collagenase
13591995	Matrix metalloproteinase-14
13592045	Rhodopsin kinase
13592047	Aminopeptidase B
148747375	Phosphatidate cytidylyltransferase 1
13786140	Cadherin EGF LAG seven-pass G-type receptor 3
13786164	Cadherin-1
13786192	Apelin receptor
13928706	Neural cell adhesion molecule 1
162287173	Carnitine O-palmitoyltransferase 1, liver isoform
13928766	Ephrin type-A receptor 3
13928822	ATP-sensitive inward rectifier potassium channel 10
13929058	CD166 antigen
13929062	Vitamin K-dependent gamma-carboxylase
13929072	Bile salt export pump
13929110	V-type proton ATPase subunit S1
13929136	Deformed epidermal autoregulatory factor 1 homolog
13994179	Sodium/potassium/calcium exchanger 2
13994184	Mannosyl-oligosaccharide glucosidase
14091742	Contactin-associated protein 1
14091750	Estradiol 17-beta-dehydrogenase 12
16258817	Copper-transporting ATPase 1
16758006	Homer protein homolog 2
16758046	C-terminal-binding protein 2
16758144	Melanoma-associated antigen D1
16758152	C-X-C chemokine receptor type 3
16758224	Neurabin-1
158186631	Cyclic nucleotide-gated cation channel alpha-4
16758274	Peroxiredoxin-4
16758300	Ectonucleotide pyrophosphatase/phosphodiesterase family member 1
16758310	Low-density lipoprotein receptor-related protein 3

16758316	Phosphatidylinositol 5-phosphate 4-kinase type-2 beta
16758342	Membrane-bound transcription factor site-1 protease
16758372	Dipeptidase 1
16758422	Membrane-associated guanylate kinase, WW and PDZ domain-containing protein 2
16758534	Embigin
16758540	CLIP-associating protein 2
168823431	Neurexin-3-alpha
16758684	Retinal guanylyl cyclase 2
16758694	Atrial natriuretic peptide receptor 2
16758746	Contactin-4
16758758	Protein ERGIC-53
16758818	Potassium voltage-gated channel subfamily H member 6
16758824	Serine/threonine-protein kinase MARK1
16758872	Cadherin-17
164663795	Potassium voltage-gated channel subfamily B member 2
16758938	AP-2 complex subunit mu
16758964	Homer protein homolog 3
16924000	Leucine-rich repeat-containing protein 7
17105342	Nasal embryonic luteinizing hormone-releasing hormone factor
17105346	Phospholipid scramblase 1
17865345	Cadherin-23
17978453	Amyloid beta A4 precursor protein-binding family B member 1
18034783	Canalicular multispecific organic anion transporter 2
18158449	Coatomer subunit beta
18266702	Vesicle transport protein SEC20
18426832	Interleukin-23 subunit alpha
18543357	Sodium-coupled neutral amino acid transporter 4
18959256	Interleukin-24
18959272	Potassium voltage-gated channel subfamily KQT member 2
19424160	Potassium-transporting ATPase alpha chain 2
19424304	Neuronal acetylcholine receptor subunit beta-3
19424324	Potassium voltage-gated channel subfamily H member 5
189083698	Caveolin-1
158138568	Serum albumin
19705437	Ephrin type-A receptor 7
19705483	Calsyntenin-2
19923689	Disks large-associated protein
19923703	CD44 antigen
19924085	Protocadherin Fat 3
20302049	NADH-cytochrome b5 reductase 3
20302083	General receptor for phosphoinositides 1-associated scaffold protein
20302091	Phospholipase B1, membrane-associated
20302101	Odorant-binding protein

20376816	Glutamate [NMDA] receptor subunit 3B
158631200	Casein kinase I isoform delta
20806147	CD48 antigen
20806161	Probable G-protein coupled receptor 116
21245116	Death domain-containing membrane protein NRADD
21955128	Leucine-rich glioma-inactivated protein 1
23618893	Leucine zipper putative tumor suppressor 1
25140983	ERC protein 2
25282419	Calnexin
25453392	Alpha-1B-glycoprotein
25742766	Fibroblast growth factor 9
26023947	Neuropilin-1
27465533	Unconventional myosin-Ie
27545388	ATP-binding cassette sub-family A member 5
27753972	Frizzled-5
27819651	Nicastrin
28212250	Voltage-dependent calcium channel subunit alpha-2/delta-2
28461145	Agrin
28570188	Chloride intracellular channel protein 6
29293825	Interleukin-1 receptor accessory protein-like 1
158186621	Complement C4
29789269	Glutamate receptor 1
29789275	Platelet-derived growth factor receptor beta
30017437	Neuronal membrane glycoprotein M6-a
163659913	Glutamate receptor, ionotropic kainate 3
31077142	Kv channel-interacting protein 4
31126963	Submandibular gland secretory Glx-rich protein CA
31542029	Multidrug resistance-associated protein 1
31542335	Voltage-dependent calcium channel subunit alpha-2/delta-1
139948535	UDP-N-acetylglucosamine--peptide N-acetylglucosaminyltransferase 110 kDa subunit
37693510	Bone marrow stromal antigen 2
38454276	Immunoglobulin superfamily member 10
39930507	Potassium channel subfamily K member 15
40254721	Amphoterin-induced protein 2
40254761	Tyrosine-protein kinase Fgr
40254779	Ephrin-B1
40385885	Follicle-stimulating hormone receptor
40538742	ATP synthase subunit alpha, mitochondrial
40538874	Nexilin
40786487	Protein GPR108
40786493	Pannexin-3
41053837	Glutathione peroxidase 3
41054820	Chordin-like protein 1

148747472	Mitofusin-2
189083700	Caveolin-1
41529837	Junction plakoglobin
42476116	Fibulin-5
42538984	Xin actin-binding repeat-containing protein 2
46048609	Catenin beta-1
46485389	Kin of IRRE-like protein 1
47059500	Neuron-specific protein family member 1
47087081	Abhydrolase domain-containing protein 16A
50511316	Jouberin
50657390	Beta-secretase 2
51036661	Type-1A angiotensin II receptor
51854227	Gelsolin
51948494	Integrin beta-6
52138521	Ezrin
52345439	Neurexophilin-4
53759105	Complement component receptor 1-like protein
54020664	Decorin
54312088	Plasma membrane calcium-transporting ATPase 4
55741439	Oncostatin-M
55741681	Integral membrane protein 2B
56090309	Myelin protein zero-like protein 1
56090341	Uncharacterized protein C1orf56 homolog
56090608	Protein CLN8
56605670	Leucine-rich repeat-containing protein 59
56605720	Leucine-rich repeat-containing protein 59
56605746	Transmembrane protein 97
56676360	Dihydroorotate dehydrogenase (quinone), mitochondrial
56797757	Fibrinogen alpha chain
57164113	Sterol-4-alpha-carboxylate 3-dehydrogenase, decarboxylating
57526957	Aspartoacylase-2
57528269	Abhydrolase domain-containing protein 14A
57977323	2',3'-cyclic-nucleotide 3'-phosphodiesterase
58865696	Uncharacterized aarF domain-containing protein kinase 4
58865778	Dolichyl-diphosphooligosaccharide--protein glycosyltransferase 48 kDa subunit
58865948	Cyclic AMP-responsive element-binding protein 3-like protein 2
60097910	Probable G-protein coupled receptor 19
161783809	Apolipoprotein B-100
61556826	NEDD4 family-interacting protein 1
61557025	ER lumen protein retaining receptor 2
61557037	NADH-cytochrome b5 reductase 1
61889129	3 beta-hydroxysteroid dehydrogenase/Delta 5-->4-isomerase type 4
62078583	Phospholipase B-like 1
62078625	Ankyrin repeat domain-containing protein 46

62078677	Coiled-coil domain-containing protein 47
62078695	Malectin
62078741	Coagulation factor XII
348605241	Kazrin
62078929	Coiled-coil domain-containing protein 51
62078985	Nucleolar complex protein 4 homolog
62079009	Transmembrane protein 192
62079201	Transmembrane protein 55B
62122956	GRAM domain-containing protein 1A
62945342	Lymphocyte transmembrane adapter 1
66730471	Transmembrane protein ENSP00000340100 homolog
67010011	C-type mannose receptor 2
67078456	Leucine-rich repeat-containing protein 8A
68163381	Protein FAM26E
68163431	Leucine-rich repeat-containing protein 33
71043602	G-protein coupled receptor 4
71043856	B-cell linker protein
71361635	Protein OSCP1
71795652	Solute carrier family 25 member 38
71896592	Insulin-like growth factor-binding protein complex acid labile subunit
75832132	Extended synaptotagmin-1
75992946	Brevican core protein
76257394	Serine/threonine-protein kinase MRCK beta
76496479	Carcinoembryonic antigen-related cell adhesion molecule 1
76559925	Calumenin
76563950	Calpain 11
77176454	Cell surface glycoprotein MUC18
77404238	Gamma-aminobutyric acid type B receptor subunit 1
77861906	Presqualene diphosphate phosphatase
77917608	Protein FAM134B
78097112	ATPase family AAA domain-containing protein 1
78365244	Alpha-adducin
81295329	Leucine-rich repeat-containing protein 8C
83816939	Alpha-1-inhibitor 3
84370262	Fibronectin type III domain-containing protein 1
86515432	Cytospin-A
88853561	Gephyrin
88853835	Formin-binding protein 1-like
6981324	Protein disulfide-isomerase
6981332	Plasminogen activator inhibitor 1
6981402	cGMP-dependent protein kinase 2
6981430	Prostaglandin-H2 D-isomerase
6981432	Prostaglandin E2 receptor EP1 subtype
6981480	Proto-oncogene tyrosine-protein kinase ROS

6981486	Dolichyl-diphosphooligosaccharide--protein glycosyltransferase subunit 1
6981494	Class I histocompatibility antigen, Non-RT1.A alpha-1 chain
6981574	SPARC
347800746	Serine protease inhibitor A3K
6981626	Protachykinin-1
6981654	Thy-1 membrane glycoprotein
6981660	Lamina-associated polypeptide 2, isoform beta
6981686	Tubby protein homolog
148747528	Protein-tyrosine kinase 2-beta
8393450	Golgi apparatus protein 1
8393944	Proenkephalin-A
8394307	Neutral and basic amino acid transport protein rBAT
8394364	Translocon-associated protein subunit delta
8394496	Tyrosine-protein kinase receptor TYRO3
9506833	Small conductance calcium-activated potassium channel protein 2
9506891	4F2 cell-surface antigen heavy chain
9507085	Neuronal-specific septin-3
9507127	SRC kinase signaling inhibitor 1
9507169	Synaptotagmin-3
9507235	2-hydroxyacylsphingosine 1-beta-galactosyltransferase
11024652	Transforming growth factor beta-1
11067413	Adenylate cyclase type 10
11177880	Vesicle-associated membrane protein-associated protein B
11968080	Protein kinase C zeta type
12083665	Serine/threonine-protein kinase TAO2
12408318	Protein unc-13 homolog A
12408320	Protein unc-13 homolog B
124107592	Unconventional myosin-Ic
12831221	Vesicle transport through interaction with t-SNAREs homolog 1A
13540638	Endoplasmic reticulum aminopeptidase 1
13591864	Neurosecretory protein VGF
13592148	Ras-related protein Rab-11A
13786160	Solute carrier family 22 member 8
13786200	Voltage-dependent anion-selective channel protein 1
13928974	Dolichyl-diphosphooligosaccharide--protein glycosyltransferase subunit 2
13929006	Ras-related protein Rab-2A
13929012	Serine protease HTRA1
13929122	Sperm-associated antigen 4 protein
13929144	Trophoblast glycoprotein
13994177	Clathrin coat assembly protein AP180
14091758	Synaptojanin-2
14861868	Receptor-type tyrosine-protein phosphatase V
15011857	Selenoprotein P
16306470	Regulating synaptic membrane exocytosis protein 1

16758080	Collagen alpha-2(I) chain
16758312	Secreted frizzled-related protein 4
16758368	Ras-related protein Rab-14
16758600	Regulator of G-protein signaling 14
16758640	Phosphatidylinositide phosphatase SAC1
16758812	B(0,+)-type amino acid transporter 1
16758874	Ras-related protein Rab-28
17939356	Synaptotagmin-like protein 4
18034781	GTP-binding protein Rab-3D
18093098	P2X purinoceptor 2
18543347	Polypeptide N-acetylgalactosaminyltransferase 10
18677747	Taste receptor type 1 member 3
18959222	Sodium channel protein type 9 subunit alpha
254675174	Urocortin-2
19424330	Mitochondrial 2-oxodicarboxylate carrier
19705557	Pancreatic secretory granule membrane major glycoprotein GP2
20127390	RING finger protein 112
399124780	Phosphate carrier protein, mitochondrial
21326477	Receptor-interacting serine/threonine-protein kinase 3
21717663	GTP-binding protein Rhes
307746876	Alpha-1-macroglobulin
23097356	Anion exchange protein 4
27465561	Sphingosine-1-phosphate lyase 1
154146247	Trimeric intracellular cation channel type A
158711704	Collagen alpha-1(I) chain
157786694	Polymerase I and transcript release factor
28212216	Trace amine-associated receptor 8a
260166629	Serine/threonine-protein kinase WNK4
28212260	Afadin- and alpha-actinin-binding protein
257796229	Ras GTPase-activating protein SynGAP
32189350	ADP/ATP translocase 2
32189355	ADP/ATP translocase 1
32563565	Serine protease inhibitor A3L
33414515	PX domain-containing protein kinase-like protein
158186720	Solute carrier family 12 member 3
158749620	Microtubule-associated protein 1B
157824216	Ras-related protein R-Ras
189163506	DnaJ homolog subfamily C member 10
158138559	Glia-derived nexin
157823984	Erlin-2
38454238	Ras-related protein Rab-15
40018584	Protrudin
41235797	SH3 and multiple ankyrin repeat domains protein 2
47058988	Mitochondrial import receptor subunit TOM70

47059002	Electrogenic sodium bicarbonate cotransporter 4
51871603	Suppressor of tumorigenicity 7 protein
51921383	SH3 and multiple ankyrin repeat domains protein 2
51948448	Ras-related protein Rab-21
52138628	Ras-related protein Rap-1b
52851375	Transient receptor potential cation channel subfamily M member 8
54114993	Ras-related protein Rap-1A
56090245	T-complex protein 11 homolog
56090369	Thioredoxin-related transmembrane protein 2
56090383	Transmembrane protein 43
56711254	Collagen alpha-1(III) chain
120474989	Keratin, type II cytoskeletal 1
57528337	Transmembrane emp24 domain-containing protein 9
58219524	Transmembrane protein 17
162135934	Protein-tyrosine sulfotransferase 1
58866008	Transmembrane channel-like protein 5
60223059	Ribonuclease-like protein 10
61676217	Signal recognition particle receptor subunit beta
61889071	Ras-related protein Rab-10
62079075	Ventricular zone-expressed PH domain-containing protein homolog 1
110624761	Protein YIPF5
187960160	Serine/threonine-protein kinase BRSK1
209977101	Transient receptor potential cation channel subfamily M member 4
46240860	Deleted in malignant brain tumors 1 protein
157821375	Phosphatidylserine synthase 2
124248495	Chitinase domain-containing protein 1
209870097	NACHT, LRR and PYD domains-containing protein 6
157822043	Plasminogen receptor (KT)
157821237	secreted frizzled-related protein 5 precursor
300798518	FCH domain only protein 2
195973006	Pikachurin
157819267	Oligosaccharyltransferase complex subunit OSTC
157787099	Neurogenic locus notch homolog protein 1
189303567	Patatin-like phospholipase domain-containing protein 7
158081781	Niban-like protein 1
282154817	secretory phospholipase A2 receptor precursor
158138549	Nck-associated protein 1
306482632	BPI fold-containing family B member 4
291045290	Probable N-acetyltransferase CML2
392347640	von Willebrand factor
281485586	Mitogen-activated protein kinase kinase kinase 7
293359269	Transmembrane protein 245
121949752	Delta(24)-sterol reductase
155369285	HEAT repeat-containing protein 8

347300280	Calsyntenin-1
158081747	Platelet-derived growth factor subunit B
117647204	T-cell surface glycoprotein CD3 gamma chain
158186672	E3 ubiquitin-protein ligase NEDD4
392350152	Disintegrin and metalloproteinase domain-containing protein 10
392350222	Collagen alpha-1(XII) chain
392349911	Amyloid-like protein 2
268607712	Sushi, nidogen and EGF-like domain-containing protein 1
158186685	Ras-related protein Rab-12
293351536	Sterol regulatory element-binding protein 1
281182792	Serine/threonine-protein kinase 10
158187531	Noggin
281306775	Lactase-phlorizin hydrolase
293352958	PREDICTED: GPI ethanolamine phosphate transferase 2 isoform 3
281306793	Ephrin type-A receptor 5
158187548	Merlin
155369293	Adipocyte enhancer-binding protein 1
157822133	Vinculin
201066363	Lysyl oxidase homolog 2
157819677	Sarcolemmal membrane-associated protein
109503020	Nischarin
281182586	sema domain, immunoglobulin domain (Ig), short basic domain, secreted, (semaphorin) 3G precursor
157819005	Ectonucleotide pyrophosphatase/phosphodiesterase family member 6
125630382	Microtubule-associated tumor suppressor 1 homolog
157823771	Hippocampus abundant transcript-like protein 1
157823803	Docking protein 3
157786758	Epididymal secretory glutathione peroxidase
281332166	Probable G-protein coupled receptor 158
209954628	Pro-neuregulin-2, membrane-bound isoform
155369271	cAMP-dependent protein kinase catalytic subunit alpha
258679494	Butyrophilin-like protein 2
209954854	Graves disease carrier protein
253970435	Guanine nucleotide-binding protein G(s) subunit alpha isoforms short
70608161	Receptor expression-enhancing protein 4
71795631	Inactive rhomboid protein 1
74271849	Sodium-dependent neutral amino acid transporter SLC6A17
77020289	Receptor tyrosine-protein kinase erbB-2
77404421	3-oxo-5-alpha-steroid 4-dehydrogenase 1
77695930	Syntaxin-12
77993298	Translocon-associated protein subunit alpha
398650622	Sodium/calcium exchanger 1
84781733	E3 ubiquitin-protein ligase UBR4
8393073	Leukocyte antigen CD37

157819501	cleft lip and palate associated transmembrane protein 1
187937124	transmembrane protein 126B
157787012	SLAM family member 9 precursor
29293815	serologically defined colon cancer antigen 8 homolog
29789369	receptor-type tyrosine-protein phosphatase gamma
148747388	thyrotropin releasing hormone receptor 2
148747388	thyrotropin releasing hormone receptor 2
157818549	transmembrane emp24 domain-containing protein 6 precursor
157818549	transmembrane emp24 domain-containing protein 6 precursor
197313797	mitochondrial carrier homolog 1
157822673	heparan sulfate glucosamine 3-O-sulfotransferase 5
157823653	Clavesin-2
157816949	nodal modulator 1 precursor
197313795	metaxin-1
121583768	ras-related protein Rab-5B
157823877	cytoskeleton-associated protein 4
157818077	EMILIN-1
157822511	T-cell surface glycoprotein CD3 epsilon chain precursor
121583784	semaphorin-3B precursor
387157894	dystroglycan precursor
194473626	cytochrome c-1
157817887	cholesterol 24-hydroxylase
157816925	pinin
274327782	desert hedgehog precursor
347300461	alkB, alkylation repair homolog 5
157823385	SLIT and NTRK-like protein 6 precursor
157786724	charged multivesicular body protein 6
114326232	glycerol-3-phosphate acyltransferase 6
312922379	tenascin-N precursor
34881169	PREDICTED: sphingolipid delta(4)-desaturase DES1
158508580	translocase of inner mitochondrial membrane 17 homolog B
157823972	PRA1 family protein 2
38454230	V-type proton ATPase subunit E 1
47087124	collagen alpha-2(XI) chain precursor
50657392	SPARC-related modular calcium-binding protein 1 precursor
51854233	protocadherin-7 precursor
56961650	kallistatin precursor
58585238	transient receptor potential cation channel subfamily M member 2
58866030	XK-related protein 5
61097926	tyrosine-protein kinase RYK precursor
61557085	spectrin beta chain, brain 1
61557316	CMP-N-acetylneuraminate-beta-galactosamide-alpha-2,3-sialyltransferase 1
62078999	TRAF3-interacting JNK-activating modulator
157823407	ralBP1-associated Eps domain-containing protein 1

197387067	IGF-like family receptor 1
112984482	suprabasin isoform 2 precursor
290563809	dermokine precursor
157819397	B-cell receptor CD22 precursor
291084648	netrin-5 precursor
157822391	otogelin precursor
157823439	tight junction protein ZO-1
157820063	alpha-mannosidase 2x
210032529	ras GTPase-activating-like protein IQGAP1
157820747	neuromedin-B precursor
157820309	zinc finger protein 592
300797871	signal peptidase complex subunit 2 homolog
157817478	phosphatidylinositol-4-phosphate 3-kinase C2 domain-containing subunit alpha
187937018	inositol 1,4,5-triphosphate receptor-interacting protein-like 2
157821515	type I inositol-1,4,5-trisphosphate 5-phosphatase
157821347	plakophilin-3
157817531	transmembrane protein 2
300794610	phosphoglucomutase-like protein 5
157823129	E3 ubiquitin-protein ligase MARCH5
157818177	cone cGMP-specific 3',5'-cyclic phosphodiesterase subunit alpha'
209364574	heparanase-2 precursor
157821237	secreted frizzled-related protein 5 precursor
157820241	MARVEL domain-containing protein 1
157822261	NADH dehydrogenase [ubiquinone] 1 beta subcomplex subunit 8, mitochondrial
157817716	AP-3 complex subunit beta-1
312836829	A disintegrin and metalloproteinase with thrombospondin motifs 12 precursor
274323706	cadherin-10 precursor
300794741	fibronectin type III domain-containing protein 3B
350534782	FRAS1-related extracellular matrix protein 2 precursor
157823757	periostin precursor
157818917	lipopolysaccharide-responsive and beige-like anchor protein
157818737	probable histone-lysine N-methyltransferase ASH1L
300798730	nuclear pore membrane glycoprotein 210-like
157819073	AP-4 complex subunit beta-1
158749598	leucine-rich repeats and immunoglobulin-like domains protein 2 precursor
157819343	fibronectin type III domain-containing protein 7 precursor
189011598	calcium-binding mitochondrial carrier protein SCaMC-1
157823739	hippocampus abundant gene transcript 1
166091462	guanylate binding protein 5
157818955	dolichyldiphosphatase 1
157822303	protein GPR107 precursor
282154817	secretory phospholipase A2 receptor precursor
109468286	integrin alpha-6 precursor
157820047	catenin delta-1

158819029	mitochondrial carrier homolog 2
208973288	phosphatidylinositol 3,4,5-trisphosphate-dependent Rac exchanger 1 protein
157822663	ras-related protein Rab-22A
300798041	laminin subunit alpha-5 precursor
201023302	semaphorin-3C precursor
157820449	krev interaction trapped protein 1
157822901	protein phosphatase 1 regulatory subunit 3A
392347666	PREDICTED: ovostatin homolog
188595692	regulator of G-protein signaling 20
157820551	alpha-catulin
293359401	SH3-containing GRB2-like protein 3-interacting protein 1
157787147	TEK tyrosine kinase, endothelial
157822407	microtubule-associated serine/threonine-protein kinase 2
187960053	ephrin type-B receptor 2 precursor
157818937	von Willebrand factor A domain-containing protein 5B1
157818937	von Willebrand factor A domain-containing protein 5B1
157822929	ephrin type-A receptor 2 precursor
300796048	arylacetamide deacetylase-like 3
300796087	pleckstrin homology domain-containing family H member 2
291167742	putative polypeptide N-acetylgalactosaminyltransferase-like protein 1
281332208	pecanex-like protein 1
300796288	SCY1-like protein 2
330340424	transmembrane and TPR repeat-containing protein 2 precursor
194474018	R-spondin-2 precursor
294345428	brain-specific angiogenesis inhibitor 1 precursor
189011703	ER lumen protein retaining receptor 3
157821883	plexin-B2 precursor
157824146	integrin alpha-5
157824172	nck-associated protein 1-like
300795929	beta-galactosidase-1-like protein 2
197927176	dolichyl-diphosphooligosaccharide--protein glycosyltransferase subunit STT3A
157823041	integrin alpha-11 precursor
124249068	carbonic anhydrase 12 precursor
157823279	cingulin-like protein 1
157817390	cell cycle progression protein 1
157819015	collagen alpha-1(VII) chain precursor
157818047	tyrosine-protein kinase-like 7
157822789	multidrug resistance-associated protein 7
300796855	partitioning defective 3 homolog B
157818183	von Willebrand factor C domain-containing protein 2-like precursor
157817097	2-acylglycerol O-acyltransferase 1
274323760	receptor-type tyrosine-protein phosphatase mu precursor
312147379	laminin subunit alpha-1 precursor
157818431	periplakin

157818689	TBC1 domain family member 9B
157819627	follistatin-related protein 4 precursor
300793998	protein shisa-6 homolog precursor
157820169	ubiquitin carboxyl-terminal hydrolase 32
157786652	vacuolar protein sorting-associated protein 53 homolog
392351689	PREDICTED: ATP-binding cassette sub-family A member 9
157819479	probable G-protein coupled receptor 128
157820883	protein FAM3B
300793816	probable cation-transporting ATPase 13A5
157786986	solute carrier family 35 member F5
201023377	SLIT-ROBO Rho GTPase-activating protein 2
157786960	phosphatidylinositol-4-phosphate 3-kinase C2 domain-containing subunit beta
281371494	laminin, gamma 2 precursor
300794137	T-lymphocyte surface antigen Ly-9
300794158	inactive phospholipase D5
281371337	laminin, beta 3 precursor
310688889	plexin-A2
281427143	protein Shroom3
157819815	trans-2,3-enoyl-CoA reductase-like
166157526	RELT-like protein 1 precursor
281182916	stromal interaction molecule 2 precursor
201066369	leucine-rich repeat LGI family member 2 precursor
157822319	limbin
198386353	myosin-Ig
157823349	serine/threonine-protein kinase VRK2
157822363	protocadherin-17 precursor
281182586	sema domain, immunoglobulin domain (Ig), short basic domain, secreted, (semaphorin) 3G precursor
300794315	leucine-rich repeat, immunoglobulin-like domain and transmembrane domain-containing protein 2 precursor
157819823	FCH domain only protein 1
157823299	chondroitin sulfate N-acetylgalactosaminyltransferase 1
300798600	EF-hand domain-containing family member A2
300794338	macrophage scavenger receptor types I and II
157819609	tumor necrosis factor ligand superfamily member 13B
157823803	Docking protein 3
300797015	constitutive coactivator of PPAR-gamma-like protein 1
157822513	engulfment and cell motility protein 1
291190715	integrin alpha-8
281371353	interleukin 17B precursor
157817201	neuropilin and tolloid-like protein 1 precursor
157153636	kinesin-like protein KIFC3
300797038	hedgehog-interacting protein precursor
157818225	contactin-associated protein-like 4 precursor

157821063	tubby-related protein 1
157823944	sushi domain-containing protein 2 precursor
157823691	sparc/osteonectin, cwcv and kazal-like domains proteoglycan 2
157821033	A disintegrin and metalloproteinase with thrombospondin motifs 14
224450992	LIM and senescent cell antigen-like-containing domain protein 1
157819029	solute carrier family 35 member F1
157819593	G-protein coupled receptor 143
157818513	protein FAM123B
157818411	probable G-protein coupled receptor 174
157818305	probable G-protein coupled receptor 101
157820859	sperm acrosome-associated protein 5 precursor
66730395	tyrosine-protein kinase TXK
67078500	transmembrane protein 5
68341983	Williams-Beuren syndrome chromosome region 17 homolog
68342011	UDP-N-acetyl-alpha-D-galactosamine:polypeptide N-acetylgalactosaminyltransferase-like 5
158631175	receptor-type tyrosine-protein phosphatase kappa precursor
76443685	surfeit 4
78097106	transmembrane protein 69
81158091	protocadherin gamma-A9 precursor
83415090	ras-related protein Rab-2B
306922410	splicing factor, arginine/serine-rich 7
84781743	probable palmitoyltransferase ZDHHC8
